# Screening of potential microbial markers for lung cancer using metagenomic sequencing

**DOI:** 10.1002/cam4.5513

**Published:** 2022-12-08

**Authors:** Qiang Chen, Kai Hou, Mingze Tang, Shuo Ying, Xiaoyun Zhao, Guanhua Li, Jianhui Pan, Xiaomin He, Han Xia, Yuechuan Li, Zheng Lou, Li Zhang

**Affiliations:** ^1^ Tianjin Chest Hospital Tianjin China; ^2^ Tianjin Medical University Tianjin China; ^3^ Hugobiotech Co., Ltd. Beijing China

**Keywords:** bronchoalveolar lavage fluid, disease typing biomarkers, lung cancer, lung microbiome, metagenomic next‐generation sequencing

## Abstract

**Introduction:**

Lung cancer is the most prevalent cancer with high mortality in China, and it is associated with the dysbiosis of the lung microbiome. This study attempted to screen for specific microorganisms as potential biomarkers for distinguishing benign lung disease from lung cancer.

**Methods:**

Bronchoalveolar lavage fluid (BALF) sample was selected in the study instead of saliva to avoid contamination with oral microorganisms, and microbial taxonomic and functional differences in BALF samples from patients with lung cancer and those with those from patients with benign lung diseases were performed based on metagenomic next‐generation sequencing, for the first time, so that microorganisms other than bacteria could be included.

**Results:**

The results showed that the intrasample diversity of malignant samples was different from benign samples, and the microbial differences among malignant samples were smaller, with lower microbial diversity, significantly changed microbial abundance and metabolic functions. Metabolic function analysis revealed amino acid‐related metabolism was more prevalent in benign samples, whereas carbohydrate‐related metabolism was more prevalent in malignant samples. By LEfSe, Metastat and Random Forest analysis, we identified a series of important differential microorganisms. Importantly, the model combining five key genera plus one tumor marker (neuron‐specific enolase) as indicators presented the optimal disease typing performance.

**Conclusion:**

Thus results suggest the value of these differential microorganisms enriched in tumors in mechanism research and may be potential new targets for lung cancer therapy. More importantly, the biomarkers identified in this study can be conducive to improve the clinical diagnosis of lung cancer and have good application prospects.

## Graphical abstract

Flow chart of this study.

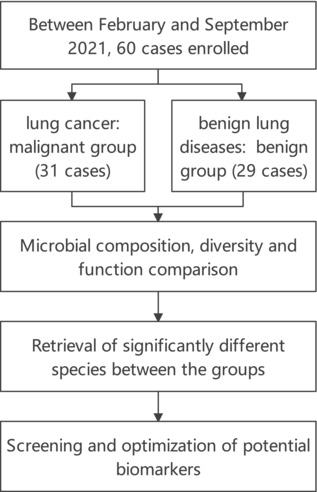

## 1 INTRODUCTION

Lung cancer is the most frequent malignant tumor across all cancer types in China with calculated 816,563 new cases and 714,699 deaths in 2020 as declared by global tumor statistics.^1^ Although the intensive research efforts and progress have obtained on the diagnosis and treatments for lung cancer, unsatisfactory prognosis and high mortality still exist, due to higher metastasis and delayed diagnosis, especially in patients with advanced stages.^2^, ^3^ Therefore, it is clinically imperative to search for novel and effective diagnostic tools to early diagnose the onset of lung cancer.

Substantial evidence has demonstrated that microorganisms are implicated with human health, affecting multiple physiological processes including nutrition metabolism, immune defense, brain activity, and disease regulation.^4^ The associations of microorganisms with metabolic diseases, autoimmune diseases, neurological disease, and even cancers have drawn lots of attentions.^4^, ^5^ Emerging studies have indicated that microorganisms exert a vital role in the occurrence, development, and treatment of cancers through regulating tumor microenvironment, predicting cancer risk, triggering inflammation to stimulate immune response, and combining with anti‐cancer drugs to enhance cancer treatment.^6^ Statistically, approximately 13% of malignant tumors are attributable to microorganisms in 2018, including *Helicobacter pylori*, hepatitis B virus (HBV), Epstein–Barr virus (EBV), and human papilloma virus (HPV).^7^ Especially, as one of the largest surface area mucosal organs in the body, the lung has been proven to possess a unique microbial community in both healthy and pathological conditions.^8^ The altered lung microbiome may contribute to tumorigenesis by changing the stability of the host genome via secretion of bacterial toxins, disrupting local immune barriers, and releasing oncogenic microbial metabolites.^9^

Given the close relationship between lung cancer and microbes, some researchers have been contributed to identify microbes associated with lung cancer as biomarkers for diagnostic purposes by leveraging revolutionary advances in culture‐independent sequencing technology.^10^ For example, Yu et al. have reported higher level of *Thermus* and lower level of *Ralstonia* in tumor tissue from patients with advanced lung cancer compared with non‐malignant lung tissue, suggesting an important role for these bacteria in lung cancer progression.^11^ Yan et al. have shown that the relative abundances of *Veillonella* and *Capnocytophaga* are markedly elevated in saliva samples from lung cancer patients.^12^ In addition, Lee et al. have also detected an incremental abundant of *Veillonella* and *Megasphaera* in bronchoalveolar lavage fluid (BALF) specimens from lung cancer patients.^13^ Moreover, Cameron et al. have demonstrated that *Granulicatella adiacens* and several other opportunistic pathogens are more frequently found in spontaneous sputum samples from lung cancer patients.^14^ Notably, previous studies screening microbial markers for lung cancer were largely investigated using 16S rRNA sequencing, while the studies based on metagenomic sequencing are limited. Therefore, the further comprehensive understanding on the diagnostic value of specific microorganisms for lung cancer using metagenomic sequencing and its role in tumor progression are still urgently necessary based on metagenomic sequencing.

Generally, the microbiota in patients with lung cancer may vary depending on the sample type, sampling method, and patient cohort. Since saliva and sputum specimens are vulnerable to oral microbial interference, and lung tissue samples are usually difficult to acquire from patients with advanced lung cancer.^15^ Therefore, in this study, the comparisons of microbial classification and functional differences between BALF specimens from lung cancer patients and patients with benign lung disease were performed to screen potential microbial markers for lung cancer based on metagenomic sequencing. Furthermore, considering tumor markers such as carcino‐embryonic antigen (CEA), neuron‐specific enolase (NSE), and cytokeratin (CYF21‐1) are widely used to evaluate the diagnosis, progression, and prognosis of lung cancer patients,^16^ these three tumor markers combined with specific microorganisms were utilized to establish the clinical prediction models for lung cancer and then evaluate the performance to improve the clinical diagnosis of lung cancer.

## 2 MATERIALS AND METHODS

### 2.1 Patient and sample collection

Between February and September 2021, patients who developed suspicious nodules based on thoracic computed tomography examination and underwent clinical bronchoscopy in Tianjin Chest Hospital were initially recruited in this study. Our inclusion criteria were as follows: nodule size of 0.8–3.0 cm on CT of the chest, single nodule, no calcified foci, no obvious satellite foci, and the presence of lymphadenopathy in the mediastinal window. If there was lymphadenopathy of ≤1.5 cm in diameter, there should be no obvious damage to the ribs or vertebrae on CT. In conclusion, diseases that could not be distinguished benign or malignant on imaging and were not associated with infection were selected. Exclusion criteria were as follows: excluding patients with acute lung infections, second primary tumors, other pulmonary comorbidities including chronic obstructive pulmonary disease, pulmonary fibrosis and bronchiectasis, or patients who had received antibiotic therapy within 1 month.

On this basis, the patients were then divided into lung cancer as malignant group and benign lung diseases as benign group according to the pathological diagnosis. We used the pathological diagnosis of specimens obtained by bronchoscopy as the gold standard and included those patients with non‐neoplastic, non‐infectious disease in the benign disease group. In fact, sarcoidosis was most common in the benign group, which is characterized by a non‐caseating necrotic granuloma indistinguishable from malignant neoplasm on imaging.

Considering the influences of several factors, including age, gender, BMI, and smoking on individual's microbiota, no statistically significant differences were ensured in these factors between the two groups, minimizing their interference with the final sequencing results. Ultimately, a total of 60 patients were enrolled in this study, including 29 cases with lung cancer and 31 with benign lung diseases.

BALF was performed in the lung on the side of the suspected nodule according to a standardized protocol developed to reduce oral contamination,^17^ and then 2 ml of BALF was collected from each patient and stored at −80°C within 30 min. In addition, the bronchoscope was washed with 10–20 ml of sterile 0.9% saline prior to the bronchoscopy, and the washing fluid was stored in sterile centrifuge tubes as negative control (NC). Lastly, a total of 90 BALF samples were obtained in this study, including 30 NC samples, 31 benign samples, and 29 malignant samples.

Both demographic and clinical characteristics were documented for all participants, including age, gender, BMI, smoking history, smoking index, tumor markers (CEA, NSE, and CYF21‐1), pathology type, and tumor stage. This study was approved by the Medical Ethics Committee of Tianjin Chest Hospital (ethical number: 2021YS‐024‐01), and the subjects' written informed consents were obtained.

### 2.2 Extraction of genomic DNA and metagenomic sequencing

Genomic DNA was extracted from BALF samples using the QIAamp DNA Microkit kit (QIAGEN). Next, the QIAseq™ Ultralow Input Library Kit for Illumina (QIAGEN) was utilized to construct the DNA libraries. The quality of the constructed libraries was assessed using Qubit fluorescence quantitative analyzer (Thermo Fisher, MA, USA) and Agilent 2100 bioanalyzer (Agilent Technologies). Finally, qualified DNA libraries were amplified and sequenced by a Nova6000 PE150 platform sequencer (Illumina).

### 2.3 Data processing and analysis

Raw data quality was assessed using FastQC software. Reads with low quality (*Q* < 30) and short fragments (<35 bp) as well as adapter contamination were removed from the raw data by Trimmomatic software, followed by the removal of human host reads through mapping data to human reference database using Bowtie2 software. The remaining reads were devoted to perform microbiota taxonomic diversity and the relative abundance calculation, respectively, using kraken2 and bracken softwares. Finally, contaminated species that defined as reads per million (RPM) > 50 in blank control and RPM ≥one‐third of the total RPM of NC at species or genus level were eliminated.

### 2.4 Statistical analysis

All statistical data analyses were performed using R software (version 3.6.1). The top 20 genera and species in relative abundance in all BALF samples were screened for the following analysis. The correlation of these genera and species in abundance between samples was assessed using Spearman's correlation analysis. The alpha diversity that reflecting the microbial diversity within a single sample was described using Chao1, Shannon, Gini–Simpson indices, and then these indices of all BALF samples were compared between the benign and malignant groups. Beta‐diversity was evaluated using principal component analysis (PCA) and non‐metric multidimensional scaling (NMDS) cluster analysis that describing dissimilarity in overall microbial community composition between the two groups, and then multi‐response permutation procedure (MRPP) was used to analyze whether the inter‐group difference in microbial community composition was significant. LEfSe and Metastat analysis were conducted to determine microorganisms that were significantly different between the two groups.

Furthermore, metagenome‐based metabolic pathway analysis was performed using HUMAnN3 software.^18^ Low‐quality and host‐filtered reads were matched to the UniRef90 gene family database, and then regrouped into MetaCyc pathways and KO categories for pathway annotation. MetaCyc abundance and KO abundance were normalized using CPM, and the differences in metabolic pathways between the benign and malignant groups were compared by Wilcoxon rank sum test, with *p* < 0.05 considered significant. Afterwards, GSVA as a non‐parametric, unsupervised method^19^ was used to perform the KEGG pathway enrichment analysis based on the normalized KO abundance (CPM), and the differences between the two groups were analyzed using the limma package in R, with the adjusted *p* < 0.05 by Bonferroni–Holm method and |log2FC| > 0.2.

Lastly, based on 40 genera with significant differences in relative abundance as well as three tumor markers (CEA, NSE, and CYF21‐1), random forest classification analysis was used to construct a genus‐based classifier that was able to discriminate malignant and benign lung diseases, using the leave‐one‐out cross‐validation method due to the small sample size,^20^ and the optimal classifier was evaluated using the R package caret. The ROC curve analysis was then conducted to assess the forecasting performance of classifier.

## 3 RESULTS

### 3.1 Baseline demographic and clinical characteristics of participants

Demographic and clinical data of 60 participants, including 29 patients with lung cancer and 31 patients with benign lung diseases, are summarized in Table S1. No significant differences were found in terms of age, gender, body mass index (BMI), smoking status, or smoking index between the benign and malignant groups. The levels of three tumor markers CEA, NSE, and CYF21‐1 displayed significant differences between the two groups. In addition, the pathology types of these 29 patients with lung cancer consisted of 12 with lung adenocarcinoma, 11 with lung squamous cell carcinoma, and six with small cell lung cancer, along with different tumor stage.

### 3.2 Microbial composition and diversity of BALF samples in patients with lung cancers and benign lung diseases

**FIGURE 1:**
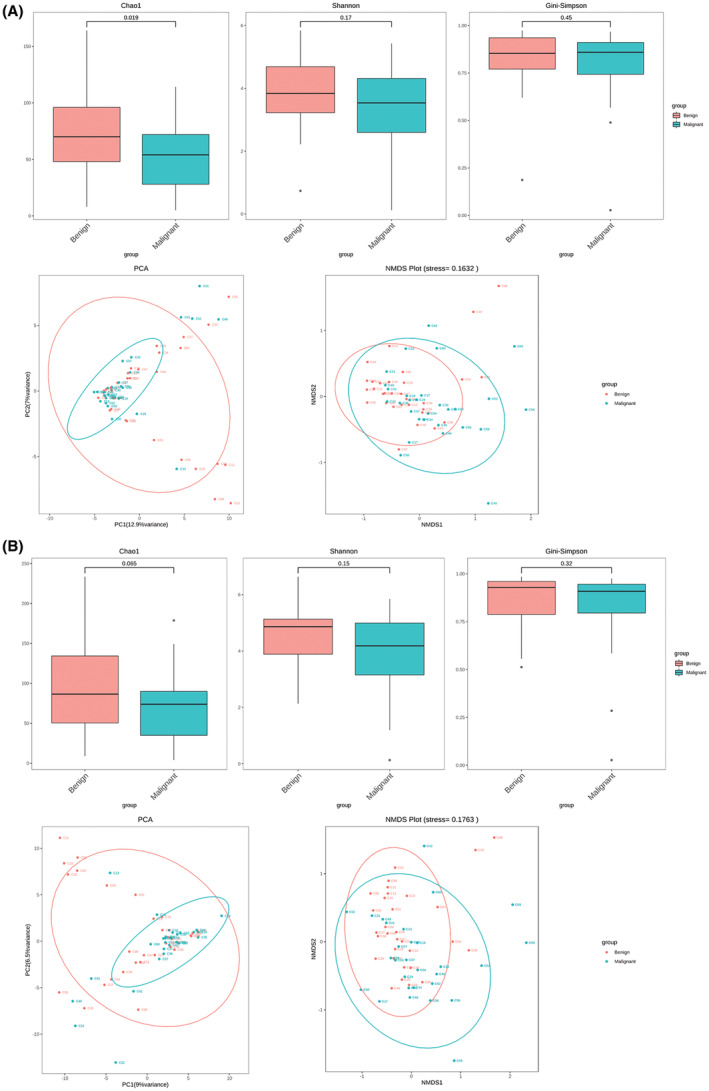
Comparison of microbial diversity of bronchoalveolar lavage fluid (BALF) between the lung cancer and benign lung diseases. (A) Comparison of alpha‐diversity of BALF sample, including Chao1, Shannon and Gini–Simpson indices, and comparison of beta‐diversity of BALF sample using principal component analysis (PCA) and non‐metric multidimensional scaling (NMDS) cluster analysis at genus levels between the malignant and benign groups. (B) Comparison of alpha‐ and beta‐diversity of BALF sample at species levels between the malignant and benign groups. Each *p* value in alpha comparison was calculated by the Wilcoxon rank sum test.

The top 20 genera and species with the highest relative abundance in all BALF samples from patients with lung cancers and benign lung diseases were screened and then combined, resulting in the pooling of 159 genera and 288 species for subsequent analysis. The correlations of these genera and species between samples were presented as a heatmap (Figure S1). Multi‐response permutation procedure (MRPP) analysis, shown in Tables S2 and S3, suggest that at genus level, the intergroup differences between benign disease and malignant tumors were significantly greater than the intragroup differences. In addition, Chao1, Shannon, and Gini–Simpson indices were then employed to measure the alpha diversity of microbial community, and the results showed that these indices of BALF samples in the malignant group was generally lower than those in the benign group but without significant differences both at the genus and species levels, excepting only for the Chao1 index at genus level that illustrated significantly difference between the two groups (Figure 1). Furthermore, both the results of principal component analysis (PCA) and non‐metric multidimensional scaling (NMDS) clustering analysis showed that there was no clear separation in microbial community both at the genus and species levels between the malignant and benign groups, while the PCA results showed that compared with benign samples, malignant samples were more clearly clustered, and NMDS results showed the opposite.

### 3.3 Metabolic pathways of BALF microbiota in patients with lung cancers and benign lung diseases

**FIGURE 2:**
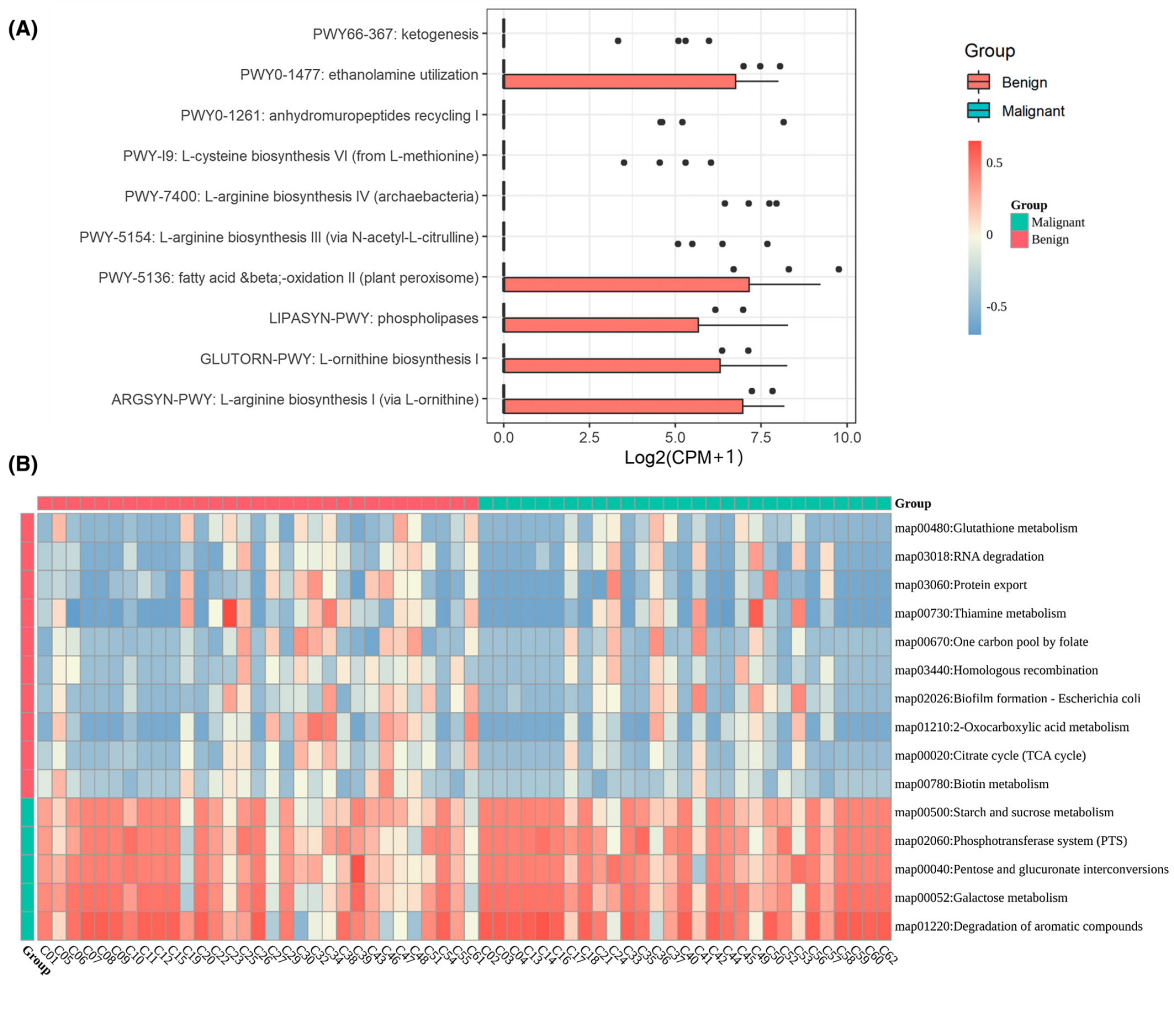
Metagenome‐based metabolic pathway analysis of bronchoalveolar lavage fluid (BALF) in patients with lung cancers and benign lung diseases. (A) Box plot of metabolic differential pathways as analyzed by MetaCyc pathway differential analysis based on HMP Unified Metabolic Analysis Network. Red color and green color, respectively, indicate log_2_ (CPM + 1) values for benign disease and malignant tumor samples. (B) KEGG differential pathways with adjusted *p* < 0.05 and |log_2_FC| > 0.2 based on Gene Set Vairant Analysis. The rows are pathways, and the columns are samples. Columns are grouped by sample type and rows are grouped by log_2_FC score of the pathway, where log_2_FC < −0.2 is the pathway with high score in the benign group, while log_2_FC >0.2 is the pathway with high score in the malignant group.

The metabolic function of BALF microbiota was further explored using HUMAnN3 software. The MetaCyc pathway differential analysis showed that compared with the malignant group, BALF microbiota in the benign group were predominantly associated with amino acid‐related metabolism, such as ketogenesis, L‐cysteine biosynthesis VI (from L‐methionine), L‐arginine biosynthesis IV (archaebacteria), L‐arginine biosynthesis III (via N‐acetyl‐L‐citrulline), L‐ornithine biosynthesis I, L‐arginine biosynthesis I, and L arginine biosynthesis I (via L‐ornithine) (Figure 2A). Meanwhile, KEGG pathway differential analysis based on Gene Set Vairant Analysis (GSVA) revealed that glutathione metabolism, RNA degradation, and protein export were significant enriched in the benign group; in contrast, carbohydrate‐related pathways were more prevalent in the malignant group, such as starch and sucrose metabolism, pentose and glucuronate interconversions, and galactose metabolism (Figure 2B).

### 3.4 Species composition characteristics in different types of cancer tissues

The top 10 genera and species in the abundance of benign disease samples and malignant tumor samples were taken, respectively, and aggregated to draw stacked maps (Figure S2). The results showed that the common microbial genera in the benign and malignant groups included *Veilonella*, *Toxoplasma*, *Streptococcus*, *Sphingobium*, *Rothia*, *Prevotella*, *Neisseria*, *Mycobacterium*, *Moraxella*, *Klebsiella*, *Haemophilus*, *Gordonia*, *Corynebacterium*, and *Bradyrhizobium*, and common microbial species included *Toxoplasma gondii*, *Staphylococcus epidermidis*, *Staphylococcus aureus*, *Rothia mucilaginosa*, *Prevotella melaninogenica*, *Prevotella jejuni*, *Prevotella intermedia*, *Neisseria subflava*, *Neisseria mucosa*, *Moraxella catarrhalis*, *Klebsiella pneumoniae*, *Gordonia polyisoprenivorans*, *Corynebacterium matruchoti*, and *Acidovorax* sp. KKS102.

### 3.5 Retrieval of significantly different species in the benign and malignant groups

**FIGURE 3:**
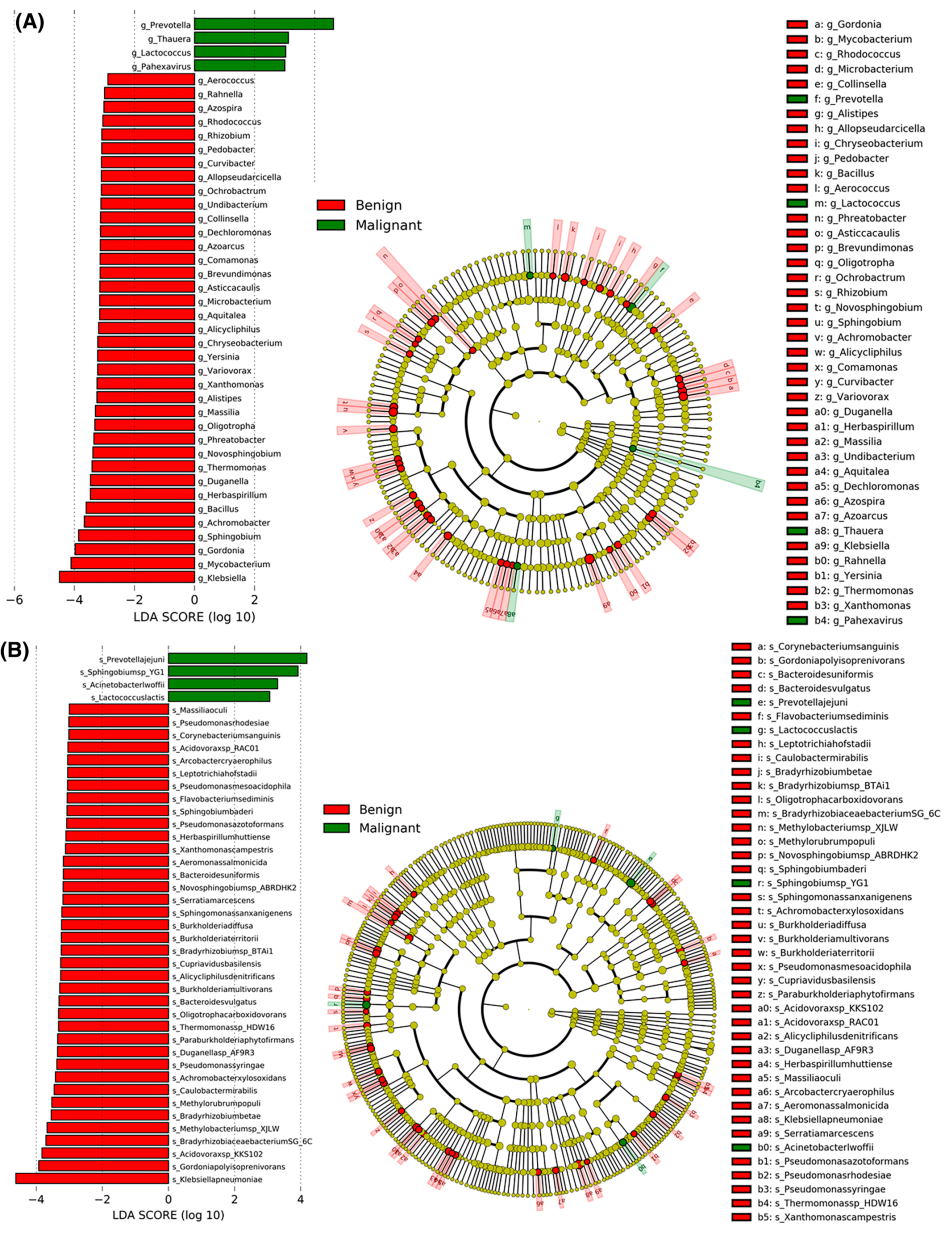
Differentially enriched microorganisms in bronchoalveolar lavage fluid (BALF) of patients with lung cancers and benign lung diseases retrieved by linear discriminant analysis (LDA) effect size (LEfSe). (A) Differentially enriched microorganisms at genus level with LDA score greater than 2 displayed by the bar chart on the left. The larger the LDA value, the greater the impact on the corresponding group and the higher the abundance. Differentially enriched microorganisms at genus level displayed by the clade diagram on the right, with circles radiating from the inside out, representing the taxonomic levels from kingdom to species. Each small circle at a different taxonomic level represents the taxonomy at that level, and the diameter of the small circle is proportional to the relative abundance. Microorganisms with no significant differences are uniformly colored in yellow, and genera with significant differences have been marked as green or red. (B) Deferentially enriched microorganisms at species level displayed by the bar chart (left) and the clade diagram (right).

Two methods, namely linear discriminant analysis (LDA) effect size (LEfSe) and Metastat, were conducted to search for differentially enriched microorganisms in BALF samples between the malignant and benign groups. LEfSe analysis was performed based on the Wilcoxon or Kruskal–Wallis rank sum test and LDA, and the genera and species with statistically significant differences in relative abundance between the two groups (threshold value with LDA score > 2) are shown in Figure 3, such as *Prevotella*, *Klebsiella*, *Mycobacterium*, *Gordonia*, and *Sphingobium* at genus level, as well as *Prevotella jejuni*, *Sphingobium* sp. YG1, *Klebsiella pneumoniae*, *Gordonia polyisoprenivorans*, and *Acidovorax* sp. KKS102 at species level. Metastat analysis, unlike LEfSe analysis, automatically adjusts the statistical method according to the sample. Metastat analysis results revealed that the top 10 genera with the most significant differences in relative abundance in BALF samples between the two groups were *Achromobacter*, *Chryseobacterium*, *Herbaspirillum*, *Pedobacter*, *Thermomonas*, *Undibacterium*, *Caulobacter*, *Novosphingobium*, *Prevotella*, and *Dechloromonas*, and the top 10 species included *Sphingobium* sp. YG1, *Streptococcus_*sp._NCTC_11,567, *Mesorhizobium_*sp._Pch‐S, *Paraburkholderia phytofirmans*, *Thermomonas* sp. HDW16, *Sphingomonas sanxanigenens*, *Methylobacterium* sp._XJLW, *Novosphingobium_*sp._ABRDHK2, *Achromobacter xylosoxidans*, and *Bradyrhizobium_*sp._BTAi1 (Table 1).

**TABLE 1:** The top 10 genera and species with the most significant differences in relative abundance in bronchoalveolar lavage fluid (BALF) between the lung cancer and benign lung diseases

OTU	Benign group (*N* = 29)	Malignant group (*N* = 31)	*p* value
Genus (mean ± SD)			
*Achromobacter*	0.000012 ± 0.000008	0.003713 ± 0.002897	0.000999
*Chryseobacterium*	0.000374 ± 0.000147	0.002942 ± 0.000739	0.000999
*Herbaspirillum*	0.000111 ± 0.000071	0.000601 ± 0.000147	0.000999
*Pedobacter*	0.000024 ± 0.000015	0.000629 ± 0.000199	0.000999
*Thermomonas*	0.001386 ± 0.000526	0.005235 ± 0.000999	0.000999
*Undibacterium*	0.000635 ± 0.000207	0.002711 ± 0.000541	0.000999
*Caulobacter*	0.000279 ± 0.000087	0.002582 ± 0.001265	0.001998
*Novosphingobium*	0.002337 ± 0.000697	0.008252 ± 0.001994	0.001998
*Prevotella*	0.189897 ± 0.037106	0.064073 ± 0.018263	0.001998
*Dechloromonas*	0.000039 ± 0.000032	0.002153 ± 0.000958	0.002997
Species (mean ± SD)			
*Sphingobium* sp. YG1	0.000934 ± 0.000352	0.010177 ± 0.003966	0.000999
*Streptococcus* sp. NCTC 11567	0.000063 ± 0.000063	0 ± 0	0.000999
*Mesorhizobium* sp. Pch‐S	0.001249 ± 0.000386	0.005587 ± 0.001353	0.001998
*Paraburkholderia phytofirmans*	0.000024 ± 0.000024	0.000473 ± 0.000145	0.001998
*Thermomonas* sp. HDW16	0.001456 ± 0.000629	0.004612 ± 0.000787	0.002997
*Sphingomonas sanxanigenens*	0 ± 0	0.00029 ± 0.000159	0.003996
*Methylobacterium* sp. XJLW	0.001523 ± 0.000757	0.009362 ± 0.002605	0.004995
*Novosphingobium* sp. ABRDHK2	0.000235 ± 0.000166	0.002459 ± 0.00077	0.004995
*Achromobacter xylosoxidans*	0 ± 0	0.005313 ± 0.004835	0.005994
*Bradyrhizobium* sp. BTAi1	0.000103 ± 0.000049	0.001881 ± 0.000662	0.005994

### 3.6 Screening and optimization of potential biomarkers

A random forest classifier was constructed to categorically rank the importance of the differential genera. Since the microorganisms with significant differences between the two groups determined by LEfSe and Metastat methods basically overlapped, all 40 genera with significant differences (*p* < 0.05, LDA > 3) based on the LEfSe results were selected for the random forest classification analysis. Leave‐one‐out cross‐validation (LOOCV) was used, and the training and testing sets were set to 3:1, with the testing results shown in Figure S3. According to mean decrease accuracy (MDA) and mean decrease Gini (MDG) indices, five genera were considered as potential markers for identification of lung cancer, including *Klebsiella*, *Mycobacterium*, *Pedobacter*, *Prevotella*, and *Xanthomonas*, with threshold value of MDA > 5 or MDG > 2.

**FIGURE 4:**
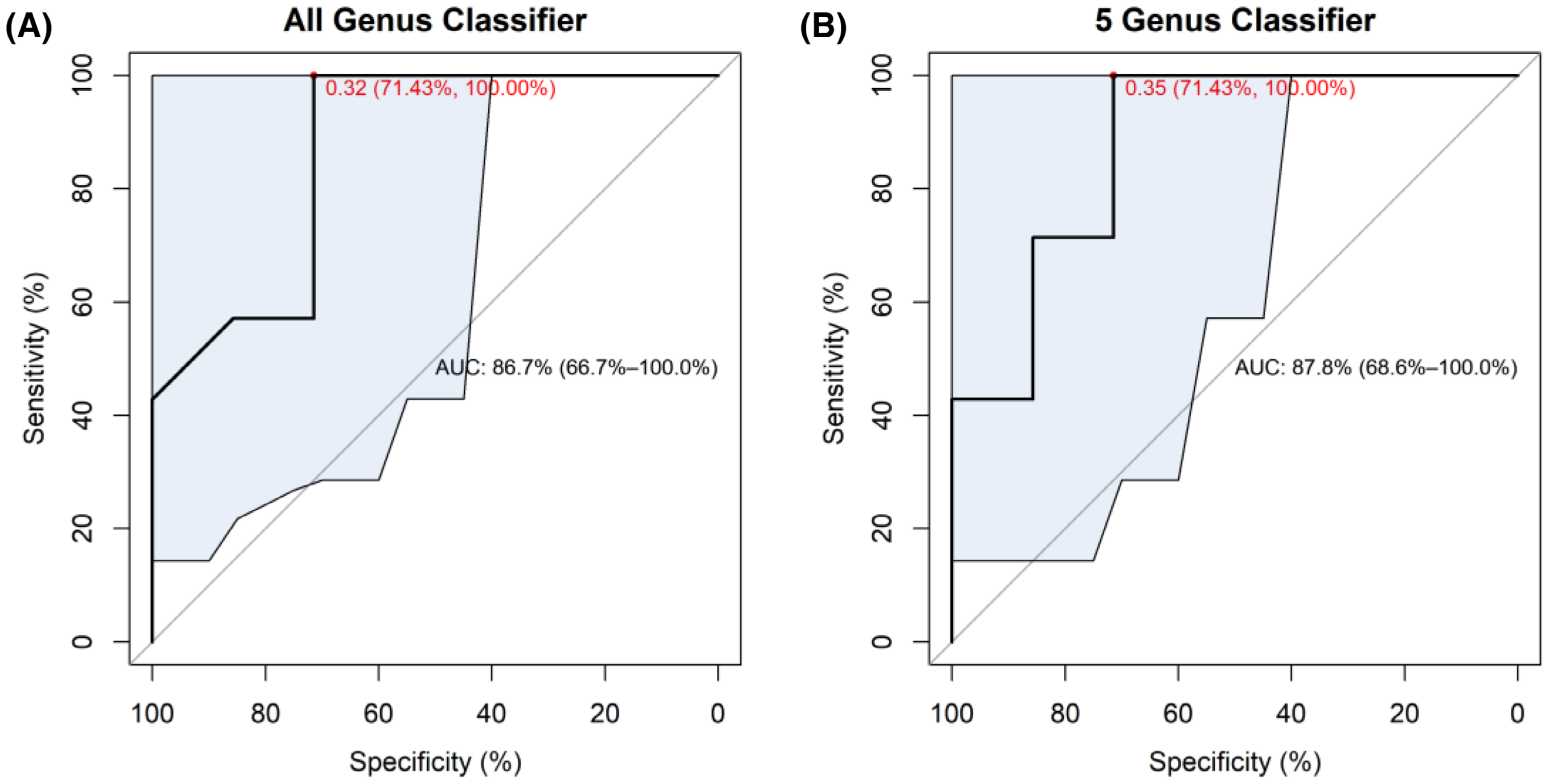
ROC curves of cancer tissue typing performance using (A) all 40 genera as biomarkers and (B) five genera as biomarkers. The shaded areas are the 95% confidence intervals of the AUC.

We further evaluated the typing ability of these five genera (Figure 4), and the results showed that using only these five genera as biomarkers was better than using all 40 genera as markers.

Considering that tumor markers CYF21‐1, CEA, and NSE can also play important roles in tissue typing, we explored what combination of microbial biomarker can achieve the highest efficiency (Figure S4).

**FIGURE 5:**
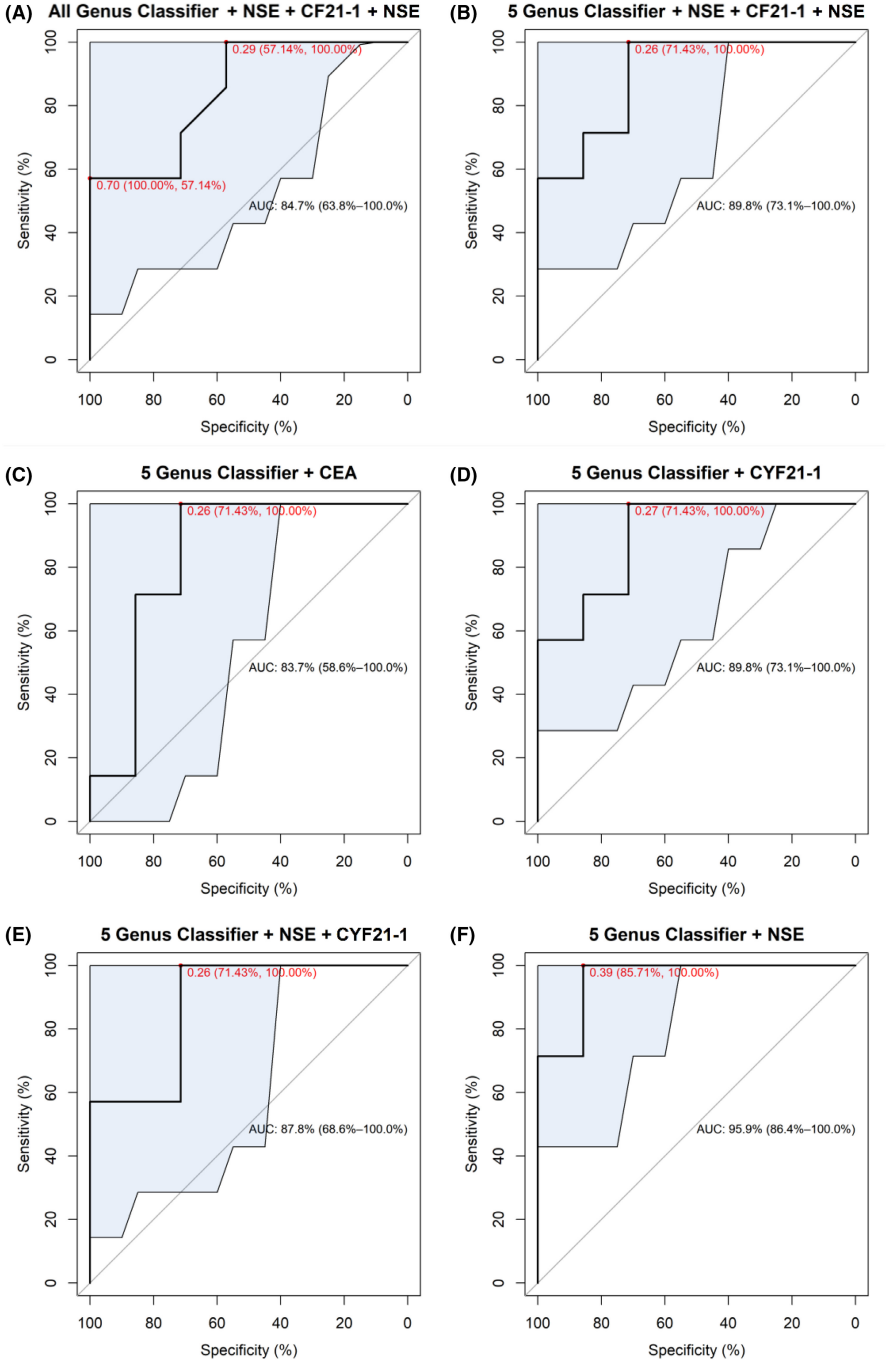
ROC curves of the classifiers of the five genus combined with three tumor markers (CEA, NSE, and CYF21‐1) for diagnosing lung cancer. A, All genus classifier + CEA + NSE + CYF21‐1. B‐F, 5 genus classifier combined with different tummarkers. The red dot represents diagnostic threshold, with larger than the threshold as malignant and lower than as benign. The data at the red dot indicates threshold (sensitivity and specificity). The shaded areas are the 95% confidence intervals of the AUC.

We further evaluated the diagnostic performance of the classifiers based on the five genera combined with three tumor markers (CEA, NSE, and CYF21‐1) in lung cancer by ROC curves (Figure 5, diagnostic performance of the three tumor markers alone shown in Figure S5). The results showed that the diagnostic performances of all genus classifier (receiver operating curve [AUC = 0.847]), five genus classifier (AUC = 0.898), five genus classifier + CEA (AUC = 0.837), five genus classifier + CYF21‐1 (AUC = 0.898), and five genus classifier + NSE + CYF21‐1 (AUC = 0.878) were similar. However, compared with the above classifiers, five genus classifier + NSE (AUC = 0.959) displayed superior diagnostic performances with 85.7% of specificity, 100% of sensitivity, and cut‐off value of 0.386.

## 4 DISCUSSION

Herein, we explored the composition of the microbiome in BALF samples of patients with lung cancer and benign lung diseases using metagenomic sequencing, as well as identified specific microorganisms that were able to serve as promising biomarkers for the early diagnosis of lung cancer. Although mNGS technology has been widely used in the clinical examination,^21^ to the best of our knowledge, it is the first time to apply it to the screening of biomarkers for lung disease typing.

A mounting number of clinical and animal experiments have suggested a link between dysregulated lung microbiome and lung cancer.^9^ It have been demonstrated that in some cancers, like cervical cancer^22^ and excised pancreatic cancer,^23^ an increase in alpha‐diversity is often linked to improved outcomes in terms of survival and treatment response, which may be realized by affecting the host's response to immunity. As previously by Lee et al., the alpha‐diversity of microbial communities varies significantly in BALF samples between the lung cancer and benign lung diseases.^13^ Similarly, our study also found that the alpha‐diversity of malignant tumors was different from that of benign diseases both at genus level and species level. In addition, beta‐diversity results showed that although no significant separation in microbial community both at the genus and species levels between the malignant and benign groups, malignant tumor group was more clustered (according to PCA analysis), representing less variation among the malignant BALF samples compared with benign samples (NMDS results showed the opposite). Furthermore, the MRPP results showed that the overall structure of the microbial communities between the two groups demonstrated a differentiation trend, which is consistent with the findings of Tsay et al.^24^ and Liu et al.^25^ that also revealed significantly different lung microbiome compositions between lung cancer and non‐malignant diseases.

The above results collectively imply that the microbiome composition in the two groups of tissues is inherently different, which is a potential prerequisite for screening microbial markers, confirming the rationality of using microorganisms as tumor markers. Although almost no microorganisms themselves are carcinogenic except some bacteria and viruses, recent studies have shown that changes in lung tissue microenvironment are related to microbial colonization,^26^ and it is possible that lower respiratory tract bacteria may participate in the occurrence and development of lung cancer through changes in lung tissue environment, and the main mechanisms are summarized as follows: (1) The activation of inflammatory pathways mediated by inflammatory microenvironment promotes the occurrence and development of lung cancer.^27^ (2) Immune microenvironment mediated immune activation promotes the occurrence and development of lung cancer in the host immune‐microbial relationship. Pulmonary microbiota induces immune tolerance through recruitment of dendritic cells, γδT, and T‐regulatory cells.^28^ (3) Metabolic regulation mediated by metabolic microenvironment promotes the occurrence and development of lung cancer. In recent years, studies have found that bacteria participate in the regulation of host metabolism, and bacteria utilize the metabolites of host cells, such as amino acids, nucleotides, polysaccharides, lipids, vitamins, etc. Meanwhile, the metabolites produced by bacteria also affect the occurrence and development of tumors, affect the growth and diffusion of tumors, inhibit cell apoptosis, and enhance tumor angiogenesis.^29^ At the same time, lung cancer is often associated with lower respiratory tract infection, which affects the composition of airway flora, affects the therapeutic effect of lung cancer, and the overall prognosis of the patient.^30^

Additionally, this study identified specific microorganisms related to lung cancer through LEfSe and Metastat analysis. The results showed significantly higher relative abundance of *Achromobacter* in the BALF samples of lung cancer patients, whereas previous studies have suggested that *Achromobacter* infection manifests as lung nodules mimicking carcinoma.^13^ Concatenation among the top 10 genera and species in the abundance of benign and malignant samples showed that the common microbial genera in each sample included *Veilonella*, which has been previously verified to be a potential biomarker for lung cancer by quantitative PCR.^12^ These differential enriched microorganisms may play specific roles in the tumor microenvironment by regulating specific metabolic pathways,^8^ Previous studies have also shown that the reduction in the abundance of specific microorganisms may increase the risk of cancer.^14^ Thus, we further conducted metagenome‐based metabolic pathway differential analysis in patients with lung cancers and benign lung diseases. The results found that amino acid‐related metabolism was more prevalent in benign samples, whereas carbohydrate‐related metabolism was more prevalent in malignant samples. It has been documented that both amino acid‐related metabolism^31^ and carbohydrate‐related metabolism^32^ are involved with the occurrence and development of various cancers. These data indicate that the inhibition of amino acid metabolism and the activation of carbohydrate metabolism may be associated with the progression of lung cancer.

To further evaluate the diagnostic performance of specific microorganisms in lung cancer, the random forest classifiers were established based on these specific microorganisms combined with three tumor markers (CEA, NSE, and CYF21‐1). According to feature importance on MDA and MDG indicators, five genera were screened as potential biomarkers, including *Klebsiella*, *Mycobacterium*, *Pedobacter*, *Prevotella*, and *Xanthomonas*. *Klebsiella* is often associated with lung infections.^33^ However, in this study, its relative abundance was significantly increased in the benign group, which may be explained that *Klebsiella* is a very common pathogenic microorganism in lung infections, and its high virulence can cause symptoms similar to those of malignant tumors, but does not lead to an increase in the incidence of malignant tumors.^34^ *Mycobacterium* is a definite pathogenic agent tuberculosis. It has been clinically proven that a history of pulmonary tuberculosis is closely associated with an increased risk of lung cancer,^35^ and blocking the PD‐1/PDL1 signaling pathway may benefit patients with *Mycobacterium tuberculosis* or other chronic infections, or even prevent their cancer development.^36^ However, related studies also pointed out that this carcinogenic effect is a long‐term effect.^37^ In this study, the higher loading of *Mycobacterium* in benign tissues seems to imply that the presence of *Mycobacterium* more predominantly marks the onset of pathogenic infection rather than lung cancer. *Pedobacter* is considered as a background microorganism in the environment.^38^ The loading of this genus in malignant sample was significantly reduced in this study, however, the relationship between *Pedobacter* and lung cancer needs to be explored further. *Prevotella* were significantly higher in lung cancer patients than in patients with benign lung disease, which is consistent with previous findings.^24^ This suggests the momentous clinical significance of the genera in lung cancer progression, which is worthy further exploration. This study has shown that in vitro exposure of airway epithelial cells to *Wolbachia*, *Prevotella*, and *Streptococcus* contribute to the upregulation of ERK and PI3K signaling pathways in lung cancer.^24^ In addition, *Xanthomonas* was significantly higher in benign samples, and consistently, microbes of this genus have been reported to play an anti‐tumor role.^39^ Afterwards, the diagnostic performance of the random forest classifiers was evaluated through the ROC curve. and we found that these five key genera + NSE presented the optimal diagnostic performance (AUC = 0.959) for lung cancer. The study of Jin et al. established a diagnostic model based on age, smoking years, and 11 types of bacteria to predict lung cancer with AUC of 0.882.^40^ Cheng et al. also constructed a random forest model similar to our study based on lung microbiome and tumor markers with AUC of 0.845 to diagnose lung cancer.^41^ Compared with the above results, the model of five genera + NSE in this study displays obvious diagnostic advantages for lung cancer.

However, our study has some limitations. First, the number of patients enrolled in this study was not large, and a larger sample size in following study is clearly beneficial for drawing more evidence‐based conclusions. In addition, lung cancer patients were not classified by histological subtypes or different stages which may cause results heterogeneity. Second, the model for distinguishing lung cancer from benign lung disease lacking evaluation of a validation cohort can lead to false‐positive values and unreliability. As a single‐center study, a multi‐center subject design should be considered to validate its findings prior to subsequent industrialization attempts. Third, this study is a cross‐sectional study that illustrates the phenomenon only from a microbiological perspective. Fourth, BALF is not as readily available as saliva, which may have limitations in clinical application. Although the possible metabolic pathways involved with lung cancer were preliminarily predicted based on the microbiome results, the interaction mechanism between them was not further explored.

## 5 CONCLUSION

In this study, we focused on the microbiome characteristics of BALF samples in patients with lung cancer and benign lung disease based on metagenomic sequencing, and predicted the metabolic pathways related to lung cancer. The model of five key differential genera + NSE established in this research exhibited remarkable performance for distinguishing lung cancer from benign lung disease. The scientific significance of these results is to highlight these specific microorganisms as potential new targets for lung cancer diagnosis and treatment, with value of mechanism research and provide directions for further in‐depth research.

## FUNDING INFORMATION

All stages of this study have received financial support from National Key R&D Project of China (No. 2019YFC0119400), and the Science and Technology Project of Xi'an (No. 21RGSF0013).

## CONFLICT OF INTEREST

The authors report no conflicts of interest. The authors alone are responsible for the content and the writing of the article.

## Supporting information


Appendix S1

## Data Availability

The datasets presented in this study can be found in National Genomics Data Center (https://www.cncb.ac.cn/), accession no. PRJCA009342.

## References

[1] Cao W , Chen HD , Yu YW , Li N , Chen WQ . Changing profiles of cancer burden worldwide and in China: a secondary analysis of the global cancer statistics 2020. Chin Med J (Engl). 2021;134(7):783‐791. 33734139 10.1097/CM9.0000000000001474 PMC8104205

[2] Bade BC , Cruz CSD . Lung cancer 2020: epidemiology, etiology, and prevention. Clin Chest Med. 2020;41(1):1‐24. 32008623 10.1016/j.ccm.2019.10.001

[3] Howlader N , Forjaz G , Mooradian MJ , et al. The effect of advances in lung‐cancer treatment on population mortality. N Engl J Med. 2020;383(7):640‐649. 32786189 10.1056/NEJMoa1916623 PMC8577315

[4] Mohajeri MH , Brummer RJM , Rastall RA , et al. The role of the microbiome for human health: from basic science to clinical applications. Eur J Nutr. 2018;57(1):1‐14. 10.1007/s00394-018-1703-4 PMC5962619 29748817

[5] Ogunrinola GA , Oyewale JO , Oshamika OO , Olasehinde GI . The human microbiome and its impacts on health. Int J Microbiol. 2020;2020:1‐7. 10.1155/2020/8045646 PMC7306068 32612660

[6] Raza MH , Gul K , Arshad A , et al. Microbiota in cancer development and treatment. J Cancer Res Clin Oncol. 2019;145(1):49‐63. 30542789 10.1007/s00432-018-2816-0 PMC11810364

[7] de Martel C , Georges D , Bray F , Ferlay J , Clifford GM . Global burden of cancer attributable to infections in 2018: a worldwide incidence analysis. Lancet Glob Health. 2020;8(2):e180‐e190. 31862245 10.1016/S2214-109X(19)30488-7

[8] Kovaleva OV , Romashin D , Zborovskaya IB , Davydov MM , Shogenov MS , Gratchev A . Human lung microbiome on the way to cancer. J Immunol Res. 2019;2019:1‐6. 10.1155/2019/1394191 PMC6710786 31485458

[9] Maddi A , Sabharwal A , Violante T , et al. The microbiome and lung cancer. J Thorac Dis. 2019;11(1):280‐291. 30863606 10.21037/jtd.2018.12.88 PMC6384374

[10] Leng Q , Holden VK , Deepak J , Todd NW , Jiang F . Microbiota Biomarkers for Lung Cancer Diagnostics. Diagnostics. 2021;11(3):407. 33673596 10.3390/diagnostics11030407 PMC7997424

[11] Yu G , Gail MH , Consonni D , et al. Characterizing human lung tissue microbiota and its relationship to epidemiological and clinical features. Genome Biol. 2016;17(1):1‐12. 27468850 10.1186/s13059-016-1021-1 PMC4964003

[12] Yan X , Yang M , Liu J , et al. Discovery and validation of potential bacterial biomarkers for lung cancer. Am J Cancer Res. 2015;5(10):3111. 26693063 PMC4656734

[13] Lee SH , Sung JY , Yong D , et al. Characterization of microbiome in bronchoalveolar lavage fluid of patients with lung cancer comparing with benign mass like lesions. Lung Cancer. 2016;102:89‐95. 27987594 10.1016/j.lungcan.2016.10.016

[14] Cameron SJ , Lewis KE , Huws SA , et al. A pilot study using metagenomic sequencing of the sputum microbiome suggests potential bacterial biomarkers for lung cancer. PloS One. 2017;12(5):e0177062. 28542458 10.1371/journal.pone.0177062 PMC5444587

[15] Whiteside SA , McGinniss JE , Collman RG . The lung microbiome: progress and promise. J Clin Investig. 2021;131(15):e150473. 34338230 10.1172/JCI150473 PMC8321564

[16] Song WA , Liu X , Tian XD , et al. Utility of squamous cell carcinoma antigen, carcinoembryonic antigen, Cyfra 21‐1 and neuron specific enolase in lung cancer diagnosis: a prospective study from China. Chin Med J (Engl). 2011;124(20):3244‐3248. 22088515

[17] Segal LN , Alekseyenko AV , Clemente JC , et al. Enrichment of lung microbiome with supraglottic taxa is associated with increased pulmonary inflammation. Microbiome. 2013;1(1):1‐12. 24450871 10.1186/2049-2618-1-19 PMC3971609

[18] Beghini F , McIver LJ , Blanco‐Míguez A , et al. Integrating taxonomic, functional, and strain‐level profiling of diverse microbial communities with bioBakery 3. Elife. 2021;10:e65088. 33944776 10.7554/eLife.65088 PMC8096432

[19] Hänzelmann S , Castelo R , Guinney J . GSVA: gene set variation analysis for microarray and RNA‐seq data. BMC Bioinformatics. 2013;14(1):1‐15. 23323831 10.1186/1471-2105-14-7 PMC3618321

[20] Ma S , Zhang F , Zhou F , et al. Metagenomic analysis reveals oropharyngeal microbiota alterations in patients with COVID‐19. Signal Transduct Target Ther. 2021;6(1):1‐11. 33986253 10.1038/s41392-021-00614-3 PMC8116522

[21] Liu D , Zhou H , Xu T , et al. Multicenter assessment of shotgun metagenomics for pathogen detection. EBioMedicine. 2021;74:103649. 34814051 10.1016/j.ebiom.2021.103649 PMC8608867

[22] Sims TT , el Alam MB , Karpinets TV , et al. Gut microbiome diversity is an independent predictor of survival in cervical cancer patients receiving chemoradiation. Commun Biol. 2021;4(1):1‐10. 33619320 10.1038/s42003-021-01741-x PMC7900251

[23] Riquelme E , Zhang Y , Zhang L , et al. Tumor microbiome diversity and composition influence pancreatic cancer outcomes. Cell. 2019;178(4):795‐806. e12. 31398337 10.1016/j.cell.2019.07.008 PMC7288240

[24] Tsay J‐CJ , Wu BG , Badri MH , et al. Airway microbiota is associated with upregulation of the PI3K pathway in lung cancer. Am J Respir Crit Care Med. 2018;198(9):1188‐1198. 29864375 10.1164/rccm.201710-2118OC PMC6221574

[25] Liu Y , O'Brien JL , Ajami NJ , et al. Lung tissue microbial profile in lung cancer is distinct from emphysema. Am J Cancer Res. 2018;8(9):1775‐1787. 30323970 PMC6176189

[26] Apopa PL , Alley L , Penney RB , et al. PARP1 is up‐regulated in non‐small cell lung cancer tissues in the presence of the cyanobacterial toxin microcystin. Front Microbiol. 2018;9:1757. 30127774 10.3389/fmicb.2018.01757 PMC6087756

[27] Akinosoglou KS , Karkoulias K , Marangos M . Infectious complications in patients with lung cancer. Eur Rev Med Pharmacol Sci. 2013;17(1):8‐18. 23329518

[28] Jin C , Lagoudas GK , Zhao C , et al. Commensal microbiota promote lung cancer development via γδ T cells. Cell. 2019;176(5):998‐1013.e16. 30712876 10.1016/j.cell.2018.12.040 PMC6691977

[29] Kim K , Kwon O , Ryu TY , et al. Propionate of a microbiota metabolite induces cell apoptosis and cell cycle arrest in lung cancer. Mol Med Rep. 2019;20(2):1569‐1574. 31257531 10.3892/mmr.2019.10431 PMC6625448

[30] Qiao D , Wang Z , Lu Y , Wen X , Li H , Zhao H . A retrospective study of risk and prognostic factors in relation to lower respiratory tract infection in elderly lung cancer patients. Am J Cancer Res. 2015;5(1):423‐432. 25628950 PMC4300720

[31] Muhammad N , Lee HM , Kim J . Oncology therapeutics targeting the metabolism of amino acids. Cell. 2020;9(8):1904. 10.3390/cells9081904 PMC7463463 32824193

[32] Peng Y , Yang H , Li S . The role of glycometabolic plasticity in cancer. Pathol‐Res Pract. 2021;226:153595. 34481210 10.1016/j.prp.2021.153595

[33] Paudel KR , Dharwal V , Patel VK , et al. Role of lung microbiome in innate immune response associated with chronic lung diseases. Front Med. 2020;7:554. 10.3389/fmed.2020.00554 PMC7530186 33043031

[34] Ding L , Yang Z , Lu J , et al. Characterization of phenotypic and genotypic traits of Klebsiella pneumoniae from lung cancer patients with respiratory infection. Infect Drug Resist. 2020;13:237‐245. 32099416 10.2147/IDR.S229085 PMC6996219

[35] Hadifar S , Mostafaei S , Behrouzi A , et al. Strain‐specific behavior of mycobacterium tuberculosis in A549 lung cancer cell line. BMC Bioinform. 2021;22(1):1‐12. 10.1186/s12859-021-04100-z PMC7992940 33765916

[36] Cao S , Li J , Lu J , Zhong R , Zhong H . Mycobacterium tuberculosis antigens repress Th1 immune response suppression and promotes lung cancer metastasis through PD‐1/PDl‐1 signaling pathway. Cell Death Dis. 2019;10(2):1‐12. 10.1038/s41419-018-1237-y PMC6362089 30718463

[37] Xiong K , Sun W , He Y , Fan L . Advances in molecular mechanisms of interaction between mycobacterium tuberculosis and lung cancer: a narrative review. Transl Lung Cancer Res. 2021;10(10):4012‐4026. 34858788 10.21037/tlcr-21-465 PMC8577982

[38] Joung Y , Jang HJ , Park M , Song J , Cho JC . Pedobacter aquicola sp. nov., isolated from freshwater. J Microbiol. 2018;56(7):478‐484. 29948823 10.1007/s12275-018-7499-3

[39] Ma J , Gnanasekar A , Lee A , et al. Influence of intratumor microbiome on clinical outcome and immune processes in prostate cancer. Cancer. 2020;12(9):2524. 10.3390/cancers12092524 PMC7564876 32899474

[40] Jin J , Gan Y , Liu H , et al. Diminishing microbiome richness and distinction in the lower respiratory tract of lung cancer patients: a multiple comparative study design with independent validation. Lung Cancer. 2019;136:129‐135. 31494531 10.1016/j.lungcan.2019.08.022

[41] Cheng C , Wang Z , Wang J , et al. Characterization of the lung microbiome and exploration of potential bacterial biomarkers for lung cancer. Transl Lung Cancer Res. 2020;9(3):693‐704. 32676331 10.21037/tlcr-19-590 PMC7354118

