# Supplementary material for: Screening of potential microbial markers for lung cancer using metagenomic sequencing
Source: Cancer Med. 2022 Dec 8;12(6):7127–39. doi: 10.1002/cam4.5513 (PMC10067086; doi:10.1002/cam4.5513)
Supplement: Supplementary file 1 — Appendix S1 [file CAM4-12-7127-s001.docx]

**Table S1.** The baseline demographic and clinical characteristics of the participants

| Variables | Malignant group (N = 29) | Benign group (N = 31) | *P* value |
| --- | --- | --- | --- |
| Age (years) | 65.41 ± 11.04 | 55.1 ± 14.2 | 0.1895 |
| Gender |  | | 0.5530 |
| Male (%) | 19 (65.52) | 18 (58.06) |  |
| Female (%) | 10 (34.48) | 13 (41.94) |  |
| BMI (kg/m^2^) | 23.22 ± 2.27 | 22.18 ± 3.10 | 0.1459 |
| Smoking status |  | | 0.4418 |
| Never smokers (%) | 12 (41.38) | 16(51.61) |  |
| Ever smokers (%) | 4 (13.79) | 6(19.35) |  |
| Current smokers (%) | 13 (44.83) | 9(29.03) |  |
| Smoking index | 411.03 ± 362.55 | 320.65 ± 388.79 | 0.3564 |
| Tumor markers |  | | |
| CEA (ng/mL) | 16.08 ± 30.53 | 2.96 ± 2.90 | 0.0287 |
| NSE (ng/mL) | 29.65 ± 29.24 | 14.51 ± 7.21 | 0.0108 |
| CYF21-1 (ng/mL) | 13.92 ± 17.06 | 3.21 ± 2.02 | 0.0022 |
| Pathology type |  | | |
| LUAD | 12 | - |  |
| LUSC | 11 | - |  |
| SCLC | 6 | - |  |
| Tumor stage |  | | |
| I | 0 | - |  |
| II | 5 | - |  |
| III | 11 | - |  |
| IV | 7 | - |  |
| Limited stage | 2 | - |  |
| Extensive stage | 4 | - |  |

BMI: body mass index; CEA: carcino-embryonic antigen; NSE: neuronspecific enolase; CYF21-1: cytokeratin 19; LUAD: lung adenocarcinoma; LUSC: lung squamous cell carcinoma; SCLC: small cell lung cancer

**Table S2.** Intergroup and intragroup differences between benign disease and malignant tumors at genus level.

| group | distance | A | Observe_delta | Expect_delta | P_value | P_adj_BH |
| --- | --- | --- | --- | --- | --- | --- |
| Malignant_vs_Benign | Bray-Curtis | 0.011800243 | 0.75039055 | 0.759351077 | 0.01 | 0.01 |

Description: Distance represents the used distance calculation method. A value greater than 0 indicates that the difference Intergroup is greater than the difference intragroup, and A value less than 0 indicates that the difference intragroup is greater than the difference Intergroup. The smaller the Observe delta value, the smaller the difference intragroup; the larger the Expect delta value. P_value indicates the P value of the hypothesis test, P_adj_BH is the P value after correction by the Benjamini-Hochberg method, if P_value or P_adj_BH is less than 0.05, it indicates a significant difference, and if P_value or P_adj_BH is less than 0.01, it indicates a very significant difference.

**Table S3.** Intergroup and intragroup differences between benign disease and malignant tumors at species level.

| group | distance | A | Observe_delta | Expect_delta | P_value | P_adj_BH |
| --- | --- | --- | --- | --- | --- | --- |
| Malignant_vs_Benign | Bray-Curtis | 0.007392 | 0.838821 | 0.845068 | 0.025 | 0.025 |

Description: Distance represents the used distance calculation method. A value greater than 0 indicates that the difference Intergroup is greater than the difference intragroup, and A value less than 0 indicates that the difference intragroup is greater than the difference Intergroup. The smaller the Observe delta value, the smaller the difference intragroup; the larger the Expect delta value. P_value indicates the P value of the hypothesis test, P_adj_BH is the P value after correction by the Benjamini-Hochberg method, if P_value or P_adj_BH is less than 0.05, it indicates a significant difference, and if P_value or P_adj_BH is less than 0.01, it indicates a very significant difference.

**Figure S1.** Heatmap of correlations between samples. A. Correlations between samples at genus level. B. Correlations between samples at species level. (The samples C01-C55 on the left side of the coordinate axis in the figure are benign disease samples, and the samples C02-C62 on the right side are malignant tumor samples).

**Figure S2** Stacked plot of species detected in each sample. A. At genus level, stacking map of the main microorganisms detected in each sample. B. Stacking map of the main microorganisms detected in each sample at species level.

**Figure S3.** According to the results of linear discriminant analysis (LDA) effect size (LEfSe), the 40 genera with the most obvious differences in BALF samples between the two groups, as well as the three tumor markers (CYF21-1, CEA, and NSE), were classified by random forest, and they were calculated based on MDA (Mean Decrease Accuracy) and MDG (Mean Decrease Gini).

**Figure S4.** According to the results of LEfSe, the 40 genera with the most obvious differences in the two groups of samples, as well as the three indicators of CYF21-1, CEA, and NSE, were classified by random forest, and they were calculated in MDA (MeanDecreaseAccuracy) and MDG (MeanDecreaseGini) feature importance on the metric.

**Figure S5.** ROC curves of cancer tissue typing performance using CYF21-1, CEA, and NSE alone. The shaded areas are the 95% confidence intervals of the AUC.
